# Biochemical components of seminal plasma and their correlation to the fresh seminal characteristics in Marwari stallions and Poitou jacks

**DOI:** 10.14202/vetworld.2017.214-220

**Published:** 2017-02-18

**Authors:** Thirumala Rao Talluri, Gorakh Mal, Sanjay Kumar Ravi

**Affiliations:** 1 Department of Animal Reproduction, Equine Production Campus, Indian Council of Agricultural Research-National Research Centre on Equines, Bikaner, Rajasthan-334001; 2 Department of Biochemistry, Biochemistry Laboratory, Indian Council of Agricultural Research - Indian Veterinary Research Institute, Palampur - 176 061, Himachal Pradesh, India

**Keywords:** alkaline phosphatase, biochemical components, lactate-dehydrogenase, Marwari, Poitou, stallion, seminal plasma

## Abstract

**Aim:**

To investigate various biochemical components of seminal plasma in Marwari stallions and Poitou Jacks and to find out their correlation with that of the seminal characteristics.

**Materials and Methods:**

In this study, semen was collected from six Marwari stallions and six Poitou jacks aged from 4 to 6 years and with known fertility status. The semen collection from the stallions were collected during the breeding season, i.e., between the months of April and June. From the collected semen ejaculates, we estimated the values of some biochemical components, viz., total protein content, total lipid content, and enzymes such as glutamic pyruvic transaminase (GPT), glutamic oxaloacetic transaminase (GOT), alkaline phosphatase (ALP), acid phosphatase (ACP), and lactate dehydrogenase (LDH) as well as concentrations of glucose, cholesterol, total calcium (Ca), and phosphorus (P) and correlations among different seminal parameters were statistically examined using the Pearson correlation coefficient.

**Results:**

In this study, we found positive correlations between semen volume as well as sperm concentration and GOT, GPT, ALP and ACP for both the group stallions. Significant correlation between motility and glucose, GOT and GPT could be an indication for their role metabolism and protection against free radicals to the spermatozoa.

**Conclusion:**

Based on the results, it is concluded that there is a positive correlation between some biochemical values such as glucose, Ca, ALP, and LDH and seminal parameters which play a key role in capacitation and onward movement of the spermatozoa.

## Introduction

Semen consists of spermatozoa suspended in a fluid medium called seminal plasma. Seminal plasma is the fluid portion of semen, secreted by both the epididymis as well as the accessory glands before and during ejaculation. Seminal plasma is a complex fluid, which serves as a vehicle for transporting ejaculated spermatozoa towards their journey from the testes to their target, the oocyte. Seminal plasma not only transports the spermatozoa but also provides protection and nutrition to the spermatozoa during their onward movement in the female reproductive tract. Seminal plasma consists of various biochemical components, such as glucose, cholesterol, proteins, metabolites, intracellular and antioxidant enzymes, mineral elements [1] which are important for sperm function and metabolism [2]. Estimation of the minerals, enzymes have been recommended as markers for semen quality since they indicate spermatozoa function and spermatozoa damage [3]. Stallion seminal plasma has an effect on the function and survival of ejaculated spermatozoa during *in* *vitro* storage and in the female genital tract, and to some extent individual stallion variation in the freezability of the semen can be explained by inherent differences in the composition of seminal plasma. Seminal factors exert negative and positive regulation in sperm capacitation, acrosome reaction, and gamete interaction. The anatomy and the secretions from accessory sex glands, their chemical composition and the functions differ between species and individuals within breed [3]. As sperm longevity and stallion fertility vary markedly between individuals, it is at most important for the equine industry to investigate the factors producing these effects.

A wide variety of biochemical components and enzymes are present in seminal plasma, but till now, the glands responsible for the production of these biochemical constituents has not yet been identified [1]. Role of various components of seminal plasma in the regulation in sperm capacitation, during acrosome reaction and gamete interaction, have been reported. For example, alkaline phosphatase (ALP) originates primarily from testis and epididymis and it can be used as a marker to differentiate azoospermia or oligospermia from ejaculatory failure in clinical cases [4]. A correlation between the activity of spermatozoa ALP release and semen quality was observed when spermatozoa undergo minimum and maximum stressful conditions in boars [5]. In the same manner, other enzymes such as glutamic pyruvic transaminase (GPT), glutamic oxaloacetic transaminase (GOT), and lactate dehydrogenase (LDH) are proved to be important for various metabolic processes which provide energy for livability, motility, and fertility of spermatozoa [6]. Hence, the estimation of the biochemical components and enzymes in seminal plasma can be recommended or indicated as biological markers for seminal quality since their levels/values indicate the sperm function, integrity and damage [7,8].

Several studies were conducted and reported about the biochemical composition of seminal plasma in different domestic livestock species [9-11]. However, limited studies have been conducted in horses and donkeys to find out the biochemical components of seminal plasma. Further, currently, available literature revealed meagre information on the composition of seminal plasma in indigenous horses and Poitou jacks. With this background, this study was designed and conducted with an objective to estimate some of the biochemical components of seminal plasma from Marwari stallions and Poitou jacks, respectively, and to correlate those with seminal attributes.

## Materials and Methods

### Ethical approval

All the experiments conducted for this study were done only after obtaining the ethical approval from the Institute’s Ethics Committee.

### Selection of animals

The semen was collected from six Marwari stallions and six Poitou jacks of known and proven fertility. Semen ejaculates were obtained using artificial vagina using estrus mare as dummy. 10 ejaculates of each animal were collected during the breeding season. All the stallions were maintained under uniform conditions of feeding and management.

### Semen collection and seminal plasma separation

This study was conducted on stallions and jacks located at Equine Production Campus, Indian Council of Agricultural Research (ICAR) - National Research Centre on Equines, Bikaner, Rajasthan. Semen ejaculates of each animal were collected during the breeding season between the months of April and June and examined spermatozoa under phase contrast microscope for motility, concentration, morphology and stained for dead sperm count. Gel portion of the semen was removed by filtration and immediately the seminal plasma was separated by centrifugation at 3000 rpm for 30 min at 4°C and stored at −20°C until further analysis. The stored seminal plasma was thawed at ambient temperature and again centrifuged at 10,000 rpm for 30 min at 4°C to remove the cell debris, if any. Biochemical parameters, viz., glucose, cholesterol, total protein, total lipid, GPT, GOT, ALP, acid phosphatase (ACP), LDH, Ca, and P were estimated from the samples by using biological kits (SPINREACT, Ark Diagnostics Pvt. Ltd., Mumbai).

### Seminal characteristics

Semen samples were collected and transported to the laboratory (37°C), within 5-10 min, and placed in a water bath at 37°C. Semen volume was estimated directly in a calibrated semen collection bottle, and sperm concentration was calculated with the help of a Neubauer hemocytometer, after dilution (1:200) of the semen sample with a 2% eosin solution. To evaluate the sperm progressive motility, a sample of the diluted semen was placed under a cover slip on a pre-warmed (37°C) slide and subjectively assessed using a phase contrast microscope. The percentage live sperm was determined by evaluating 200 sperm/semen sample (following eosin-nigrosin staining), under a light microscope. At least 200 cells were counted in duplicates for each sample.

### Plasma membrane integrity

Evaluation of the plasma membrane/functional integrity of sperm membrane was determined by hypo-osmotic swelling test (HOST) as described by Talluri *et al*. [12]. The HOST evaluates the functional integrity of the sperm’s plasma membrane and also serves as a useful indicator of fertility potential of sperm. A total of 200 spermatozoa were counted in different fields at 40× under phase contrast microscope and percentage of spermatozoa positive to HOST (having coiled tails) was determined by the evidence of plasma membrane swelling and HOS reacted (tail coiling). The number of spermatozoa (percentage) with tail coiling was recorded for each sample. The experiment was repeated five times in order to obtain a consistent result.

### Statistical analysis

The statistical analysis was performed by GLM procedure of Statistical Package for Social Sciences, Version 17.0. Pearson’s correlation coefficient test was applied to find out the correlation between various biochemical constituents and seminal attributes.

## Results

The observed mean values of various seminal parameters, viz., pH, volume, concentration, percentages (%) of live and dead spermatozoa, progressive sperm motility, abnormal spermatozoa, and HOST were measured and presented in Table-1.

**Table-1:** Mean±SD seminal attributes of fresh semen ejaculates of the Marwari and Poitou Stallions.

Seminal parameters	Mean±SD	

	Marwari stallions	Poitou jacks
pH	7.29±0.03^a^	7.30±0.026^a^
Volume (ml)	45.06±4.75^a^	64.53±7.09^a^
Concentration (×10^6^)	253.33±8.52^a^	262.33±15.89^a^
Live dead (%)	82.67±0.73^a^	85.2±0.78^a^
Progressive motility (%)	77.00±2.22^a^	81.46±0.87^a^
Total abnormalities (%)	2.07±0.27	3.27±0.85
HOST (%)	65.43±0.64^a^	59.36±1.61^a^

Means with different superscripts differ significantly (p≤0.05), among individual stallions within breed. HOST=Hypo osmotic swelling test, SD=Standard deviation

### Seminal parameters

The values for volume of the semen obtained, concentration of the spermatozoa, live dead ratio, abnormal spermatozoa and progressive motility of the spermatozoon fresh semen were found to be on higher side in donkey stallions than in horse stallions. However, HOS positive sperms were found to be more in Marwari stallions. There was no significant difference was observed.

### Biochemical profiles of the seminal plasma

The mean, median, minimum and maximum values for various biochemical components of the seminal plasma for Marwari stallions and Poitou jacks were listed in Table-2. Except for the ALP values, all other components such as total protein, glucose, total lipids, cholesterol, GOT, GPT, LDH, ACP, Ca and P levels were found be higher in the Poitou donkey stallion seminal plasma than that of the values observed in the case of Marwari stallion seminal plasma.

**Table 2:** Mean±SE biochemical attributes of semen ejaculates of the Marwari and Poitou Stallions.

Parameters	Marwari				Poitou			
	
	Minimum	Maximum	Median	Mean±SE	Minimum	Maximum	Median	Mean±SE
Glucose (mg/dl)	7.11	49.12	20.17	24.31±2.92	18.06	36.11	24.31	25.94±1.28
Total protein (g/dl)	0.70	1.76	0.84	0.95±0.04	3.21	5.32	4.13	4.33±0.12
Total lipid (mg/dl)	19.79	80.42	61.78	54.07±4.42	100.09	163.43	133.47	132.49±4.51
Cholesterol (mg/dl)	1.98	9.01	5.50	5.45±0.49	16.78	32.08	20.99	23.08±1.02
GOT (U/L)	119.78	378.98	158.32	215.31±21.46	123.56	389.81	142.14	218.46±22.18
GPT (U/L)	9.8	15.09	10.87	11.70±0.36	7.81	34.08	21.09	20.65±1.85
LDH (U/L)	460	1202	681.5	782.63±54.55	1681	2310	2238	2082.23±49.14
ALP (U/L)	6.43	40.04	9.23	18.03±2.66	8.18	16.88	10.47	11.84±0.61
ACP (U/L)	1.00	11.00	4	4.93±0.50	3	9	5	5.60±0.33
Calcium (mg/dl)	7.11	23.08	11.89	13.66±1.13	9.21	25.09	20.53	18.07±1.16
Phosphorus (mg/dl)	7.29	10.62	8.715	8.73±0.16	7.28	14.82	14.02	11.92±0.59

SE=Standard error, GOT=Glutamic oxaloacetic transaminase, GPT=Glutamic pyruvic transaminase, LDH= LDH=Lactate dehydrogenase, ALP=Alkaline phosphatase, ACP=Acid phosphatase

### Correlations between seminal parameters and biochemical components of seminal plasma

The correlation coefficients were calculated between the seminal parameters and biochemical components in both Marwari stallions and Poitou donkeys and were presented in Tables-3 and 4, respectively. We observed a positive and significant correlation between glucose and motility and between Ca and motility for both the groups. In this study, a positive correlation between the motility, concentration and HOST% with GOT, GPT and LDH levels was observed and found to be non-significant in both the studied groups. Positive correlations between semen volume as well as sperm concentration and GOT, GPT, ALP and ACP was observed for both the groups. From these results, it is assumed that these factors may have testicular origin. Significant correlation between motility and glucose, GOT and GPT was found which could be an indication for their role metabolism and protection against free radicals to the spermatozoa. ALP has showed negative correlation with volume of the semen, concentration and motility of the spermatozoa for both the groups and a nonsignificant positive correlation of Ca with pH, volume, concentration and HOST%. A significant correlation was observed between motility and Ca for both the groups.

**Table-3:** The correlation levels between enzyme activities and semen parameters in seminal plasma of Marwari stallions.

S.no	Seminal attributes and Biochemical parameters	1	2	3	4	5	6	7	8	9	10	11	12	13	14	15	16	17
1	pH	1	0.085	−0.209	−0.231	0.063	0.066	0.401	0.015	−0.392	−0.479	0.442	−0.409	0.236	0.266	0.061	0.020	0.388
2	Volume (ml)		1	0.004	0.013	0.304	0.304	0.030*	0.064	−0.450	0.243	−0.335	0.053	−0.575	−0.455	0.121	−0.589	−0.089*
3	Concentration (×10^6^)			1	−0.074	0.115	−0.232	0.401	−0.282	−0.155	0.435	0.333	0.172	0.534	−0.382	0.051	0.318	0.092
4	Dead (%)				1	−0.466	0.646	−0.086	−0.247	−0.112	−0.247	0.008	−0.322	0.112	0.259	−0.083	0.610	0.149
5	Mot (%)					1	0.262	0.285*	0.491	0.657*	0.197	0.492	0.372	0.150	−0.745	0.108	0.529*	0.383
6	HOST (%)						1	−0.058	0.311	−0.023	−0.412	0.071	0.064	0.446	0.266	−0.069	−0.790	0.059
7	Glucose (mg/dl)							1	0.114	−0.059	−0.117	0.357	−0.635*	−0.194	−0.047	0.033	−0.012	0.124
8	Total protein (g/dl)								1	0.630	−0.278	−0.104	0.153	0.178	−0.177	0.366	−0.079	0.068
9	Total lipid (mg/dl)									1	0.247	0.136	0.439	−0.113	−0.723	−0.048	0.329	−0.001
10	Cholesterol (mg/dl)										1	0.043	0.499	−0.045	−0.558	−0.250	0.373	−0.193
11	GOT (U/L)											1	0.002	−0.136	−0.598	−0.329	0.369	0.451
12	GPT (U/L)												1	0.397	−0.492	−0.292	0.214	−0.200*
13	LDH (U/L)													1	0.295	−0.429	−0.538	−0.420
14	ALP (U/L)														1	0.514	0.034	−0.151
15	ACP (U/L)															1	0.329	0.601
16	Ca (mg/dl)																1	0.204
17	P (mg/dl)																	1

HOST=Hypo osmotic swelling test, GOT=Glutamic oxaloacetic transaminase, GPT=Glutamic pyruvic transaminase, LDH=Lactate dehydrogenase, ALP=Alkaline phosphatase, ACP=Acid phosphatase, LDH=Lactate dehydrogenase

* Correlation coefficients were significant; p<0.05

**Table-4:** The correlation levels between enzyme activities and semen parameters in seminal plasma of Poitou jacks.

S.no	Seminal attributes and Biochemical parameters	1	2	3	4	5	6	7	8	9	10	11	12	13	14	15	16	17
1	pH	1	0.494	0.083	−0.019	−0.554	−0.459	−0.220	0.093	0.058	−0.032	0.304*	0.330	0.350	0.157	0.116	0.622	0.460
2	Vol (ml)		1	0.329	0.612	0.408	0.660	0.082	0.532	−0.210	0.217	0.374	0.364	−0.226	−0.039	0.031	0.013	−0.391
3	Concentration (×10^6^)			1	0.007	0.225	0.707	0.439	0.433	−0.277	−0.029	−0.91	−0.002	−0.283	−0.158	0.034	0.171	0.251
4	Dead (%)				1	−0.006	−0.297	0.455	−0.098	0.468	0.496	0.088	0.048	0.116	−0.639*	0.633	0.119	−0.260
5	Mot (%)					1	0.614*	0.455*	0.370	−0.719	0.215	0.046	0.022	0.131	−0.128	−0.349	0.573*	0.118
6	HOST (%)						1	0.322	0.609	−0.592	0.087	0.120	0.140	0.385	−0.043	−0.432	−0.307	0.034
7	Glucose (mg/dl)							1	−0.010	−0.082	0.468	−0.447	−0.110	−0.404	−0.685*	0.148	0.143	0.173
8	Total protein (g/dl)								1	−0.053	0.432	0.348	0.423	0.069	0.119	−0.368	−0.008	0.141
9	Total lipid (mg/dl)									1	0.454	0.274	0.263	−0.025	−0.051	0.440	0.748	−0.154
10	Cholesterol (mg/dl)										1	0.146	0.265	−0.096	−0.237	0.094	0.171	0.251
11	GOT (U/L)											1	0.501	0.660*	0.602	0.287	0.195	−0.289
12	GPT (U/L)												1	0.597	0.146	−0.308	0.379	0.034
13	LDH (U/L)													1	−0.143	0.146	−0.183	0.127
14	ACP (U/L)														1	−0.143	−0.317	−0.165
15	ACP NP (U/L)															1	0.459	−0.396
16	Ca (mg/dl)																1	0.194
17	P (mg/dl)																	1

HOST=Hypo osmotic swelling test, GOT=Glutamic oxaloacetic transaminase, GPT=Glutamic pyruvic transaminase, LDH=Lactate dehydrogenase, ALP=Alkaline phosphatase, ACP=Acid phosphatase

* Correlation coefficients were significant; p<0.05

## Discussion

Mammalian seminal plasma is a carrier for sperm and contains a number of factors crucial for normal fertilization. As of today, meagre information is available about the correlation between semen parameters and enzyme activities of stallion seminal plasma. Analysis of the enzyme activities and concentrations of elements can estimate integrity and function of sperm cell membranes. In this study, we attempted to quantify the different biochemical constituents present in the seminal plasma of indigenous stallions and Poitou Jacks. Biochemical estimation of various enzyme activities and minerals like Ca and P may indicate the seminal quality as they play a key role in the functional integrity and function of sperm cell membranes [13]. Therefore, knowledge of the levels of these enzymes can be used as biomarkers for semen quality since they indirectly indicate the sperm damage [7,8].

In this study, some biochemical components of seminal plasma of equines were estimated and correlated those with the seminal parameters. The data on seminal parameters obtained, in the current study, were correlating with the previous reports on Marwari stallions and Poitou jacks [14,15]. The biochemical values observed for GOT, GPT, LDH and ALP are also consistent with the earlier reports in horses [8,16]. In the current study, we observed a positive correlation between the motility, concentration and HOST% with GOT, GPT and LDH levels which is correlation with the previous reports [8,16]. A positive correlation between concentration, motility, HOST and GOT, GPT and LDH observed in the present study, indicated their role in semen quality because they measure sperm membrane stability [17]. An increase in the values of these enzyme activities indicates the damage to the functional integrity of acrosome or plasma membrane of spermatozoa and thus leading to abnormal spermatozoa, further which causes leakage of these enzymes in the extracellular fluid [18]. GOT and GPT play an important role in the protection of spermatozoa from oxidative stress and provides an indicator of a primary testicular and epididymal origin of these enzymes in stallion [19,20]. A positive correlation was observed between the GOT, GPT, ALP and ACP with seminal volume also indicate that these factors might have testicular origin.

ALP is a widely distributed phosphomonoesterase [21] enzyme and is active in many tissues, and very little information is available on its activity in the seminal plasma of stallions [4]. It was reported to be originating from seminal vesicles and to a lesser extent from the testes and epididymis in bull [22]. In a study conducted on horse seminal plasma composition, Turner and McDonnell [23] reported that the mean concentration of the ALP activity in horse ejaculates was observed to be 15.443±6391 IU/L and the values ranged from 22.180 to 3574 IU/L for horse stallions. The results obtained in the current study also consistent with these reports. In our study, ALP has showed negative correlation with volume of the semen, concentration and motility of the spermatozoa for both the groups. These results are consistent with the earlier reports of Dogan *et al*. [16] in Arabian horses and contradictory to the observation of Pesch *et al*. [8]. In dogs, ALP activity is similarly found in the epididymides and in the semeniferous tubules [24,25] and can be used as a marker for ejaculation and tubular patency [25,26]. ALP activity has been found in seminal vesicle fluid in bulls and boars, and in prostatic secretions in many mammals [27]. High levels of ACP are found in the prostatic fluid of primates and canines, and lower levels are found in bull prostate fluid. The levels of ACP activity reflect prostate function in dogs [28], and increased levels of ACP are associated with prostate cancer in humans [29-31]. In stallion, studies on ACP have been limited to epididymal tissue but not on seminal plasma [32]. The levels of both ALP and ACP in seminal plasma are positively correlated with the sperm count and negatively with semen volume in horses and they are believed to be under androgenic control [15,27]. ALP levels observed to be vary in boar seminal plasma with change in season [33]. LDH plays an important role in the sperm metabolism, sperm capacitation, and fertilization [6]. LDH activities in stallion spermatozoa were measured to be 81.0 IU/L by Pesch *et al*. [8], 13.3 µmol/ml [34] and 1.1 µmol/ml [2], whereas it was found 782.63±54.55 for horses and 2082.23±49.14 IU/L in our study (Table-1) and these results are in correlation with that of the values observed in Arabian horses [16].

Calcium is the most extensively studied components of mammalian semen and is mainly involved in fertilization process by inducing the acrosome reaction and is needed for stimulation of steriodogenesis in leyding cells of the testis [35]. Ca levels regulate capacitation and hyperactivation of spermatozoa by regulating the availability of intracellular adenosine triphosphate [36,37]. Increased levels of ionized Ca can induce acrosome exocytosis and lead to a decrease in fertility [38]. We observed a positive correlation of Ca with pH, volume, concentration, and HOST%. A significant correlation was observed between motility and Ca for both the groups.

## Conclusion

Seminal plasma is an important fluid portion necessary for metabolism of spermatozoa as well as for sperm function and survival and transport in the female genital tract. Analysis of biochemical constituents like enzyme activities and concentrations of elements can estimate integrity and function of sperm cell membranes. From the current study results, it can be said that the correlation was found between enzyme activities of seminal plasma and semen parameters in stallions, it was also observed that the composition of semen varied not only between the groups and breeds but also in the animals of same group. In part, this may have been due to seasonal variations and differences in age. Another contributing factor may have been a fluctuating ratio between the different fractions which compose the normal ejaculate that has contributed to the make-up of the whole ejaculate. This study is first of its kind to study biochemical composition of the seminal plasma of indigenous stallions.

## Authors’ Contributions

TRT conceived and designed the experiments. GM and SKR performed the experiments. TRT and SKR analyzed the data. TRT and GM wrote the manuscript.
